# Locally Acquired Eastern Equine Encephalitis Virus Disease, Arkansas, USA

**DOI:** 10.3201/eid2212.160844

**Published:** 2016-12

**Authors:** Jeremy Garlick, T. Jacob Lee, Patrick Shepherd, W. Matthew Linam, Daniel M. Pastula, Susan Weinstein, Stephen M. Schexnayder

**Affiliations:** University of Arkansas for Medical Sciences, Little Rock, Arkansas, USA (J. Garlick, T.J. Lee, P. Shepherd, W.M. Linam, S.M. Schexnayder);; Centers for Disease Control and Prevention, Fort Collins, Colorado, USA (D.M. Pastula);; University of Colorado School of Medicine, Aurora, Colorado, USA (D.M. Pastula);; Arkansas Department of Health, Little Rock (S. Weinstein)

**Keywords:** eastern equine encephalitis virus, encephalitis, alphavirus, arbovirus, viruses, pediatrics, brain edema, zoonoses, Arkansas, United States

**To the Editor:** Eastern equine encephalitis virus (EEEV) is an arbovirus (family *Togaviridae*, genus *Alphavirus*) transmitted to humans primarily from *Aedes*, *Coquillettidia*, and *Culex* mosquitoes. EEEV is maintained in a transmission cycle between *Culiseta melanura* mosquitoes and birds in freshwater hardwood swamps (*1*). Affected humans and horses are considered to be dead-end hosts; that is, they usually do not develop sufficient levels of viremia to infect mosquitoes. Although human EEEV disease is rare, it has a case-fatality rate of >30% and >50% of survivors may have permanent neurologic sequelae (*2**–**5*). Cases occur sporadically each year, primarily along the eastern and Gulf coasts of North America, but no cases have been previously reported in Arkansas (*1*). We report a locally acquired case of human EEEV disease in Arkansas.

In October 2013, a male teenager from southwestern Arkansas sought care at a local hospital after 3 days of headache and 3 new-onset focal seizures. He had a history of recent multiple mosquito bites and no history of recent travel. Initial laboratory studies on postsymptom onset day (PSOD) 3 showed normal peripheral leukocyte count, electrolytes, and liver function tests. Cerebrospinal fluid (CSF) exam showed 5 leukocytes/mm^3^ (reference 0–5), 7 erythrocytes/mm^3^ (reference 0), 55 mg/dL glucose (reference 45–80), and 36 mg/dL protein (reference 15–40). Noncontrast computed tomography (CT) of the head was normal. He was transferred to a regional academic pediatric hospital on PSOD 3.

On PSOD 4, the patient became increasingly lethargic, febrile (39.2°C), tachycardic, and hypotensive. He received intravenous fluids and broad-spectrum antimicrobial drugs (e.g., vancomycin, ceftriaxone, acyclovir, and doxycycline). Magnetic resonance imaging of the brain on PSOD 4 revealed left frontal lobe edema and multiple T2 signal abnormalities in the basal ganglia and midbrain. Repeat CSF examination on PSOD 4 showed 1,170 leukocytes/mm^3^ (74% neutrophils), 137 erythrocytes/mm^3^, 204 mg/dL protein, 66 mg/dL glucose, and a negative Gram stain. CSF bacterial cultures, CSF herpes simplex virus PCR, and CSF enterovirus reverse transcription PCR tests were negative.

Repeat CT scan of the brain on PSOD 6 showed frontal and temporal lobe edema. Physicians initiated measures to monitor and control elevated intracranial pressure, including placement of an external ventricular drain, hyperosmolar therapy with mannitol and 3% sodium chloride, cooling to 34°C, chemical paralysis, and a pentobarbital-induced coma. Pressors were subsequently added to maintain cerebral perfusion pressures >60 mm Hg (i.e., minimum for adequate brain perfusion). Despite these measures, elevated intracranial pressure (from low 20s mm Hg to mid-30s mm Hg) continued for 2 weeks. On PSOD 19, the patient’s intracranial pressure increased to 71 mm Hg. A repeat CT scan of the brain showed widespread cerebral edema, uncal herniation, intraparenchymal hemorrhages, and obstructive hydrocephalus. Given clinical worsening, the family elected to withdraw care and the patient died.

Serologic testing from PSOD day 4 for immunoglobulin (Ig) M and G antibodies against St. Louis encephalitis, West Nile, and California serogroup viruses was negative. A commercial EEEV IgM antibody immunofluorescence assay (IFA) performed on CSF collected on PSOD 4 was positive (titer 8). Confirmatory testing was performed at the Centers for Disease Control and Prevention’s Arboviral Diseases Branch (Fort Collins, CO, USA). Serum collected on PSOD 12 tested positive for EEEV IgM antibodies by microsphere immunoassay and for EEEV neutralizing antibodies by plaque reduction neutralization testing (titer >20,480) (*6*). Additional CSF collected on PSOD 12 also tested positive for EEEV IgM antibodies by microsphere immunoassay and for EEEV neutralizing antibodies by plaque reduction neutralization testing (titer 32).

Although human EEEV disease cases had been reported in neighboring Louisiana, Mississippi, and Texas, no cases had previously been reported in Arkansas (*1*). However, EEEV was identified in horses in Arkansas before 2013 and in the patient’s county of residence in 2013, indicating that the virus was already present in the area (*7*). Several freshwater swamps, which are known to be important ecologic environments in the EEEV transmission cycle, were within a 6-mile radius of the patient’s residence (*8*). This case shows that human EEEV disease can occur in areas where EEEV is circulating in the environment, highlighting the need for continued surveillance for EEEV and other arboviruses. Furthermore, the lack of a specific antiviral therapy for EEEV disease indicates the importance of mosquito-bite prevention strategies (e.g., using insect repellent and wearing long-sleeved shirts and pants outdoors).

For those who develop EEEV disease, supportive care is currently the only treatment option. Elevated intracranial pressure should be watched for, monitored, and aggressively managed. Hyperosmolar therapy, external ventricular drain placement, cooling, sedation, and paralysis have been used in the management of elevated intracranial pressure for other conditions and have been used with varying degrees of success in treating EEEV disease (*9**,**10*). Further research regarding the management of EEEV disease is needed.
